# Sodium oligomannate reduces cerebral infarction and improves neurological function through microbiota remodeling in MCAO/R rats

**DOI:** 10.3389/fphar.2026.1880590

**Published:** 2026-07-17

**Authors:** Jin Han, Wei Zhao, Rui Deng, Yu Wang, Wenping Gong, Zitong Wang, Guangqiang Sun, Hongchun Liu, Meiyu Geng, Yu Zhang

**Affiliations:** 1 School of Pharmacy, Anhui University of Chinese Medicine, Hefei, China; 2 Shandong Laboratory of Yantai Drug Discovery, Bohai Rim Advanced Research Institute for Drug Discovery, Yantai, Shandong, China; 3 State Key Laboratory of Drug Research, Shanghai Institute of Materia Medica, Chinese Academy of Sciences, Shanghai, China

**Keywords:** gut dysbiosis, ischemic stroke, MCAO/R, neuroinflammation, sodium oligomannate (GV-971)

## Abstract

**Background:**

Ischemic stroke is the second leading cause of death worldwide, characterized by high mortality and a narrow therapeutic window for thrombolysis. Gut microbiota dysbiosis and gliosis following ischemic stroke are key drivers of post‐stroke neurological impairment. Sodium oligomannate (GV‐971) is a low‐molecular‐weight acidic oligosaccharide that targets the gut–brain axis. It alleviates gliosis and improves cognitive dysfunction by remodeling gut microbiota in Alzheimer’s disease. However, it is still unknown whether GV‐971 has pharmacological activity against ischemic stroke.

**Methods:**

Here, we explore the efficacy of GV‐971 on infarct volume, gliosis, blood‐brain barrier integrity, gut microbiota composition, and post‐stroke cognitive impairment (PSCI) using a middle cerebral artery occlusion/reperfusion (MCAO/R) model in male Sprague‐Dawley rats.

**Results:**

Administer medication before surgery for 4 consecutive days and once after surgery, after stroke 24‐hour triphenyltetrazolium chloride (TTC) staining revealed that 0.3 mg/kg GV‐971 significantly reduced infarct volume in ischemic brain tissue from 37.81±2.391% to 13.30±4.801% and neurological impairment score of GV‐971 treatment significantly decreased from 11.50±0.54 to 7.29±1.47. After stroke 24‐hour immunofluorescence analysis of glial activation confirmed that GV‐971 significantly reduced central inflammatory responses. Western blot combined with Evans blue staining collectively demonstrated that after stroke 24‐hour, GV‐971 exerts a significant protective effect on the blood‐brain barrier. In the gut, GV‐971 reversed microbial dysbiosis, as revealed by shotgun metagenomics, enhanced intestinal barrier integrity, and suppressed colonic inflammation. Antibiotic depletion abolished GV‐971's neuroprotective effect, while fecal microbiota transplantation from GV‐971‐treated donors restored protection, supporting a microbiota‐dependent contribution. Furthermore, GV‐971‐treated rats subjected to MCAO/R exhibited significant improvements in motor and cognitive function. For example, on day 35, Y‐maze test results indicated that GV‐971 administered either before MCAO/R (pre‐treatment) or during the perioperative period (co‐treatment) increased spontaneous alternation rate from 60.95±4.91% to 85.60±6.32% and 85.64±5.027%. On day 32, novel object recognition assay results indicated that GV‐971 treatment increased new‐object exploration from 0.2039±0.03752 to 0.3991±0.1122 (pre‐treatment) and 0.5066±0.06982 (co‐treatment). On day 42, Barnes maze test results indicated that GV‐971 treatment reduced the time required to locate the target hole from 76.45±17.41s to 31.03±20.75 s and 33.37±19.30 s for pre‐ and co‐treatment, respectively.

**Conclusion:**

Taken together, GV‐971 demonstrated neuroprotective potential in experimental ischemic stroke.

## Graphical Abstract

**Figure:**
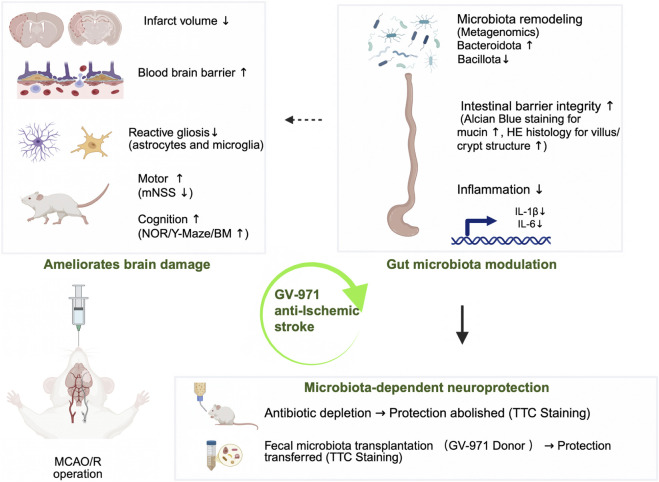

## Highlights

- GV-971 reduces infarct volume and improves neurological function in a rat MCAO/R model of ischemic stroke.
- GV-971 attenuates gliosis by suppressing glial activation and preserves blood-brain barrier integrity.
- GV-971 remodels gut microbiota, enriching beneficial taxa while suppressing pathobionts after ischemic stroke.
- Neuroprotection by GV-971 is microbiota-dependent, as efficacy is lost after antibiotics and restored by FMT.
- GV-971 prevents hippocampal neuronal loss and improves cognitive impairment in post-stroke rats.

## Introduction

Ischemic stroke is a major global health challenge, with incidence rates steadily rising worldwide. According to the Global Burden of Disease Study (GBD), ischemic stroke accounts for 60%–70% of all stroke cases globally, ranking as the second leading cause of death (An et al., 2025; GBD 2019 Stroke Collaborators, 2021; Hilkens et al., 2024). The pathophysiology of ischemic stroke involves cerebral atherosclerosis, thrombosis and embolism, which disrupt cerebral blood flow, oxygen delivery and energy metabolism, triggering ischemic-hypoxic neuronal death and subsequent neurological dysfunction.

Emerging evidence has implicated the gut-brain axis as a significant contributor to this pathological network. Cerebral ischemia activates sympathetic and hypothalamic-pituitary-adrenal (HPA) axis stress responses, which compromise intestinal and blood-brain barrier integrity, promote microbiota dysbiosis, and facilitate the translocation of pro-inflammatory mediators that exacerbate neuroinflammation and neuronal injury (Zhao et al., 2023; Benakis and Liesz, 2022; Chidambaram et al., 2022). Conversely, specific beneficial microbes and their metabolites can counteract this pathological cascade by reinforcing barrier function and modulating immune responses, thereby exerting neuroprotective effects. A study has shown that under specific experimental conditions, *Parabacterioids distason* (*P. distason*) exhibits potential neuroprotective effects, reduces reactive oxygen species (ROS) levels, inhibits the NF-κB signaling pathway, attenuates inflammatory responses, and lowers serum uric acid levels in rats with hyperuricemia associated acute ischemic stroke (Wei et al., 2024). Similarly, viable *Akkermansia muciniphila* (but not heat-killed bacteria) mitigates stroke-induced neurological impairment, reduces cerebral infarction and enhances blood-brain barrier and intestinal barrier integrity under specific conditions (Wang et al., 2025)^.^ Current pharmacological treatments for ischemic stroke primarily focus on mechanical thrombectomy, thrombolysis, anticoagulation, secondary prevention strategies, neurorehabilitation and improving cerebral perfusion (Tsivgoulis et al., 2023), with commonly used agents including alteplase (recombinant tissue plasminogen activator, rt-PA) (Tsivgoulis et al., 2023; Campbell et al., 2018)^,^ aspirin and clopidogrel (Gao et al., 2023). Although these therapies provide symptomatic relief and improve acute outcomes, they are limited by narrow therapeutic windows and cannot effectively prevent complications such as post-stroke cognitive impairment (PSCI), mechanical thrombectomy has limited applicability and rehabilitation and secondary prevention can only improve symptoms and cannot reverse brain damage, leaving substantial unmet clinical needs (Ge et al., 2024)^.^ Targeted therapies for PSCI remain particularly scarce. This gap has motivated the search for novel therapeutic strategies, with gut microbiota modulation emerging as a promising target for intervention in neurodegenerative and cerebrovascular diseases (Zhang F. et al., 2024).

Notably, the neuroinflammatory and gut-dysbiosis mechanisms underlying PSCI overlap with the therapeutic targets of Sodium oligomannate (GV-971), a low-molecular-weight acidic oligosaccharide approved for the treatment of mild-to-moderate Alzheimer’s disease (Wang et al., 2019; Liebner et al., 2018). Its primary mechanism of action involves remodeling the gut microbiota, which subsequently reduces inflammation in both the peripheral and central nervous systems, thereby alleviating neural damage and cognitive impairment. Beyond Alzheimer’s disease, GV-971 has demonstrated therapeutic potential in a range of pathological models. For instance, in severe acute pancreatitis, it impedes disease progression by modulating the gut microbiota-metabolism-immune axis to mitigate local pancreatic and systemic inflammation (Chen X. et al., 2024). Similarly, in models of vascular dementia, GV-971 improves neurological function and counteracts oxidative stress-mediated brain injury (Ren et al., 2026)^.^ *In vitro* and *in vivo* studies of synucleinopathies have further revealed that GV-971 inhibits the pathological aggregation of α-synuclein (Yu et al., 2024). Moreover, in a mouse model of neuromyelitis optica spectrum disorder (NMOSD), treatment with GV-971 delays disease progression by remodeling the gut microbiota, ameliorating peripheral inflammation and metabolic dysfunction, and reducing neuroinflammation (Yang et al., 2024)^.^

In summary, these findings indicate that GV-971 exerts anti-inflammatory and antioxidant effects across diverse disease models, primarily through the regulation of the gut microbiota and the restoration of the gut-brain axis. Given that these mechanisms align closely with the core pathophysiology of ischemic stroke, we hypothesized that GV-971 might also confer robust neuroprotection in ischemic stroke by interrupting this pathological loop. However, to date, the therapeutic efficacy of GV-971 in ischemic stroke lacks sufficient experimental validation. To address this gap, we established a middle cerebral artery occlusion/reperfusion (MCAO/R) model in Sprague-Dawley rats to recapitulate the ischemia-reperfusion injury characteristic of clinical ischemic stroke, and systematically investigated the potential neuroprotective effects and underlying mechanisms of GV-971 against ischemic stroke. Specifically, we aimed to determine: (i) whether GV-971 reduces infarct volume and improves neurological deficits; (ii) whether its neuroprotective effects require gut microbiota remodeling; (iii) whether GV-971 improves long-term cognitive outcomes after stroke.

## Materials and methods

### Reagent

GV-971 (Lot No: 220304) was obtained from Green Valley Pharmaceuticals. 3-n-Butylphthalide (NBP) was purchased from Med Chem Express (Monmouth Junction, NJ, United States; Cat. No.: HY-B0647; Assay: 99.98%), approved by the National Medical Products Administration of China for ischemic stroke therapy, 3-Butylphthalide (NBP) is chosen as the positive control in this study.

### Animals

The male Sprague-Dawley (SD) rats (240–270 g) were obtained from Beijing VTLH Laboratory Animal Technology Co. Ltd. (Beijing, China) and raised in well-ventilated cages under controlled conditions including temperature (20 °C–25 °C), relative humidity (60%), and a 12 h light/dark cycle, with unlimited access to food and water. All experimental procedures were approved by the Institutional Animal Care and Use Committee at Shanghai Institute of Materia Medica.

### Middle cerebral artery occlusion/reperfusion (MCAO/R)

The male SD rats were fasted for 12 h prior to surgery, weighing 240–270 g. The SD rats were anesthetized with Zoletil® 50 (15 mg/kg) and Xylazine hydrochlorides (5 mg/kg) intraperitoneally (i.p.) for MCAO surgery. The monofilament (251–280, MSRC37B200PK50, RWD, China) was inserted into the common carotid artery (CCA) along the external carotid artery (ECA) and then into the internal carotid artery (ICA) approximately 20 mm to the middle cerebral artery before the ECA was ligated. The wound was sutured with sterile surgical suture to reduce the bleeding, and was disinfected with iodine. The rat was placed at 37 °C on a heating pad until recovery from anesthesia. After 120 min, the monofilament was slowly withdrawn approximately 10 mm (to the marked position), and the excess monofilament was trimmed. Food and water were provided after surgery. Rats in the Sham group were manipulated using the same surgical procedure, but without monofilament insertion. Neurological function was evaluated in 24 h after surgery. Animals that died during or within 24 h after surgery due to anesthesia accidents, subarachnoid hemorrhage, or other conditions were excluded.

### Modified Neurological Severity Score (mNSS)

Neurological deficits were evaluated using the Modified Neurological Severity Score (mNSS; range 0–18), with higher scores indicating more severe impairment. Animals were evaluated 24 h after middle cerebral artery occlusion/reperfusion (MCAO/R). The mNSS comprised four domains:

Motor function (0–6 scores). Spontaneous activity/flat-walking was scored as follows: normal gait, 0 score; inability to walk straight, 1 score; circling towards the contralateral side, 2 scores; leaning towards the ipsilateral side, 3 scores. Tail suspension items were scored as follows: flexion with internal rotation and adduction of the right forelimb, 1 score; flexion with internal rotation and adduction of the right hind limb, 1 score; raising both forelimbs >10° within 30 s, 1 score.

Sensory function (0–2 scores). Visual/tactile placing: failure to place the ipsilateral forelimb on the table corner after whisker stimulation, 1 score. Proprioception: no response when the paw was placed on the cage edge and the thigh was stimulated, 1 score.

Balance beam (0–6 scores). Rats were tested on a balance beam (100–166 cm in length, 2.5 cm in width, elevated 10 cm). Performance was scored as: stable posture, 0; grasping the beam edge, 1 score; holding the beam with one limb slipping off, 2 scores; holding the beam with both limbs slipping off (>60 s), 3 scores; attempted balancing but falling within 40 s, 4 scores; attempted balancing but falling within 20 s, 5 scores; immediate fall within 20 s without an attempt to balance, 6 scores.

Reflexes and abnormal movements (0–4 scores). One point was assigned for each of the following abnormalities: impaired auricular reflex (head turning after right ear canal stimulation), 1 score; impaired corneal reflex (blinking after right conjunctival stimulation), 1 score; impaired startle reflex (response to paper-tearing sound), 1 score; and the presence of tremor, convulsions, or abnormal muscle tone, 1 score. Neurological scoring was performed by observers blinded to treatment allocation and independently verified by multiple observers.

### 2,3,5-triphenyltetrazolium chloride (TTC) staining

Brain tissues were rapidly collected and cut into 6 2-mm-thick coronal slices. The samples were stained with 1% 2,3,5-triphenyltetrazolium chloride (TTC; T8877, Sigma-Aldrich) at 37 °C for 10 min. The samples were then fixed in 4% paraformaldehyde. Images were acquired after 24 h of fixation, and infarct volume was quantified using ImageJ. In this study, infarct quantification was performed using the ischemic area ratio method. The calculation formula was as follows: ischemic volume ratio = (sum of white infarct areas across all sections)/(sum of total brain areas across all sections) × 100%.

### Drug treatment

The animals were randomly assigned to seven groups: Sham group, MCAO/R group, NBP (12.5 mg/kg) group, and GV-971 (0.03, 0.1, 0.3, and 3 mg/kg) groups. Before surgery, GV-971 was administered by intragastric gavage at a dose of 2 mL/kg once daily for three consecutive days. It was administered once 2 h before surgery and once 22 h after surgery. NBP was administered once by intravenous injection at the same dose during reperfusion (Liu et al., 2021). The Sham group was administered vehicle only. Investigators were blinded during outcome assessment.

### Hematoxylin and eosin (H&E) staining

24 h after surgery, rats were anesthetized and perfused prior to tissue collection. The colon tissue was excised, fixed, paraffin-embedded, and sectioned at 5 μm. H&E staining was performed according to the manufacturer’s instructions (C0105S, Beyotime). Images were acquired using a multispectral pathology imaging system (Vectra Polaris, United States). H&E Scoring Criteria (Forster et al., 2022)^.^ Epithelial hyperplasia/damage and goblet cell depletion: 0, no hyperplasia or damage with normal goblet cell numbers; 1, mild epithelial hyperplasia or focal damage with a slight reduction in goblet cells; 2, moderate hyperplasia or multifocal epithelial damage with marked goblet cell loss; 3, severe hyperplasia or extensive epithelial damage/shedding with near-complete absence of goblet cells. Lymphocytic infiltration of the lamina propria: 0, no appreciable inflammatory cell infiltration; 1, mild, focal infiltration; 2, moderate, diffuse infiltration with interstitial edema; 3, severe, diffuse infiltration with disruption of glandular architecture. Extent of inflammatory involvement: 0, none; 1, <25% of the tissue area affected; 2, 25%–50% affected; 3, >50% affected. Features of severe inflammation (crypt abscesses, submucosal inflammation/edema): 0, absent; 1, focal crypt abscesses or mild submucosal inflammation/edema; 2, multiple crypt abscesses or moderate submucosal inflammation/edema; 3, diffuse crypt abscesses or severe submucosal inflammation/edema. All H&E staining sections were scored by two observers who were unaware of the experimental grouping.

### Quantification of TNF-α, IL-6, and IL-1β by ELISA

Approximately 30 mg of colon tissue adjacent to the cecum was collected, thoroughly homogenized in 1× PBS until no visible sediment remained, and the supernatant was collected as the sample. A 96-well plate was coated with 100 μL per well of diluted capture antibody, sealed, and incubated overnight at room temperature. Each well was washed three times with 400 μL of 1× washing buffer, and residual moisture was removed. Then, 300 μL of reagent diluent was added to each well to block the plate surface, followed by incubation at room temperature for 1 h. After three additional washes and removal of residual moisture, the plate was ready for use. Subsequently, 100 μL of sample was added to each well, the plate was covered, and incubated at room temperature for 2 h. For the standard curve (using TNF-α as an example), the standard was reconstituted in reagent diluent according to the manufacturer’s instructions. Following concentration determination, serial dilutions were prepared in reagent diluent to obtain concentrations of 0, 62.5, 125, 250, 500, 1,000, 2000, and 4,000 pg/mL. After three washes to remove residual moisture, 100 μL of detection antibody diluted in reagent diluent was added to each well, the plate was sealed, and incubated at room temperature for 2 h. Following three washes, 100 μL of streptavidin-HRP B working diluent was added to each well, the plate was covered, and incubated at room temperature in the dark for 20 min. After three washes, 100 μL of ELISA TMB substrate was added to each well and incubated at room temperature in the dark for 20 min. Then, 50 μL of stop solution was added to each well, and the plate was gently tapped to ensure thorough mixing. For the measurement of IL-6 and IL-1β, the corresponding standard reagents and detection antibodies (streptavidin-HRP) were substituted accordingly. The optical density (OD) was measured at 450 nm using a microplate reader, with wavelength correction set at 540 nm. The OD values at 540 nm were subtracted from those at 450 nm to correct for optical defects.

### Nissl staining

Brain tissues were cut into 20 μm coronal slices and stained with Nissl reagent (Beyotime, C0117) for 10 min at room temperature. ROI was determined by rat brain stereotaxic mapping, and images were collected from the ipsilateral M1 region (Bregmar +0.48 mm) and ipsilateral hippocampal CA1 and CA2 regions (Bregmar −3.60 mm). Three coronal slices with the same coordinates were selected from the infarcted area of each animal, and three fixed area fields were selected for each slice. Neuron counting standard: Only count surviving neurons with intact cell bodies, clear Nissl bodies, and visible nucleoli. Images were acquired using a motorized inverted fluorescence microscope (IX73, Olympus) (20×). Blinding method: All slices were renumbered by a technician who did not participate in the experiment, and then manually counted by two independent observers who were unaware of the experimental grouping. Take the average of 3 fields of view for each slice, and the final result is the average of two observers’ counts, and calculate the inter observer consistency.

### Alcian blue (AB) staining

24 h after surgery, rats were anesthetized and perfused prior to colon tissues collection. Paraffin-embedded rat colon tissues were cut into 5 µm. The colon tissues were stained with Alcian blue (C0155M, Beyotime). Images were acquired using a multispectral pathology imaging system (Vectra Polaris, United States). Acidic mucopolysaccharides in goblet cells bind the cationic dye Alcian blue via ionic interactions, producing a characteristic bright-blue staining of mucin within goblet cells. The fixed threshold method of ImageJ (threshold range 170–180) was used to quantify the optical density per unit area, reflecting the total content of mucin in the organization. All slices were analyzed using the same threshold in the same batch to ensure comparability of staining intensity. Analyze 3 non consecutive slices of the same anatomical location (spaced 100 μm apart) for each animal and take the average value.

### Evans blue (EB) extravasation into brain tissue

A 2% Evans blue (EB) solution (4 mL/kg; CAS 314–13-6; Meilunbio) was administered by intravenous injection. After 2 h, each rat was perfused with saline solution and brain tissue was rapidly collected. Tissues from the injured (ipsilateral) hemisphere were collected, homogenized in 400 µL of 50% trichloroacetic acid (TCA) using a high-throughput tissue grinder (SCIENTZ-48), and centrifuged at 12,000 × g for 5 min. A 200 µL aliquot of the supernatant was transferred to a clear 96-well plate, and absorbance values at 620 nm were measured using a microplate reader. EB content was quantified by interpolation from a standard curve.

### Laser speckle flow imaging

Laser speckle flow imaging is performed 24 h after surgery to analyze the recovery of local cerebral blood flow after cerebral ischemia-reperfusion. All animals were anesthetized and skull was exposed and removed (a circle with a diameter of 1 cm). The laser speckle detector (RFLSI ZW, RWD) was positioned 10 cm above the skull to monitor cerebral blood flow, and the cerebral blood flow images were acquired and saved. This detection is not used as a validation indicator for the success of the MCAO model.

### Western blot

Brain tissue was homogenized in high-intensity RIPA lysis buffer and the samples were ground using a high-throughput tissue grinder (SCIENTZ-48) at 60 Hz for 2 min. The protein concentration was then determined using a BCA assay (ZJ101, Shanghai Yamei). Equal amounts of denatured protein were resolved by 10% SDS-PAGE and transferred to nitrocellulose membranes. After blocking, the membranes were incubated overnight at 4 °C with the primary antibodies against β-actin (S0B0005, 1:5,000), ZO-1 (21773-1-AP, 1:1,000) and Occludin (27260-1-AP, 1:1,000). The membranes were then incubated with secondary antibodies for 2 h, developed using an enhanced chemiluminescence kit (34096, Thermo Fisher Scientific), and imaged using an immunoblotting system (Touch Imager, e-blot). Densitometric analysis was performed using ImageJ, and the data were normalized to the corresponding internal loading control (β-actin) before statistical comparison among groups. Biological duplication: Tissue samples from 6 independent animals (n ≥ 6) were used in each group, with each lane corresponding to one animal, without sample mixing. The representative band shown in the picture comes from one of the samples. Data Transparency: All uncut raw membrane images, including molecular weight standard annotations, have been used as Supplementary Material.

### Immunofluorescence staining

For immunofluorescence staining, frozen coronal tissues (20 µm) from the dorsal hippocampus were washed three times in 1× PBS (5 min each). Tissues were permeabilized in 0.3% Triton X-100 for 10 min and blocked in 10% goat serum for 1–2 h, then incubated overnight with primary antibody at 4 °C. The primary antibodies included GFAP (1:800, MAB360, Merck) and Iba-1 (1:1,000, 019-1974, Wako). Tissues were then incubated for 2 h at room temperature with the secondary antibodies: Alexa Fluor 488 anti-mouse IgG (1:500, A21202, Invitrogen) and Alexa Fluor 555 anti-rabbit IgG (1:500, A31572, Invitrogen). DAPI (P0131, Beyotime) was added to the tissues and then the tissues were imaged on a laser scanning confocal microscope (FV3000, Olympus). Hippocampal images were acquired from the ipsilateral side at bregma −3.60 mm, and cortical images were acquired from the ipsilateral M1 region at bregma +0.48 mm. Three brain slices were taken from each animal at the same location, and three non-overlapping fields of view were randomly selected in the surrounding area of the infarction for each slice. All samples were randomly numbered by a technician who was not involved in animal grouping. Both image acquisition and quantitative analysis were performed by two independent observers who were completely unaware of the grouping information, and the average was taken as the final result. The quantitative indicator was the ratio of the number of GFAP positive cells and Iba-1 positive cells to the number of DAPI cells, calculated as a percentage.

### Quantitative reverse transcription PCR (qRT-PCR)

Total RNA from the colon tissue was extracted using Trizol reagent (Cat. No. 04908264, Adamas Life). The RNA concentration and purity were assessed with a NanoDrop spectrophotometer (Cat. No. 840-317500, Thermo Fisher Scientific). Reverse transcription was performed using the Evo M-MLV Reverse Transcription Premix Kit (Cat. No. AG11728, Accurate Biology), and subsequent qPCR was carried out with the SYBR Green Pro Taq HS Premix Kit (Cat. No. AG11735, Accurate Biology). The Ct values of all samples were first standardized using the internal reference gene GAPDH, and then the relative expression level of the target gene was calculated using the 2^−ΔΔCt^ method. The sequences of all primers, amplification product lengths, and annealing temperatures were listed below:

**Table:** 

Gene	Forward (5′to3′)	Reverse (5′to3′)	Amplicon (bp)	Annealing (°C)
GAPDH	TGA​TGG​GTG​TGA​ACC​ACG​AG	AGT​GAT​GGC​ATG​GAC​TGT​GG	152	60
IL-1β	CAC​CTC​TCA​AGC​AGA​GCA​CA	CGG​GTT​CCA​TGG​TGA​AGT​CA	80	60
IL-6	AGA​GAC​TTC​CAG​CCA​GTT​GC	AGC​CTC​CGA​CTT​GTG​AAG​TG	142	60
TNF-α	CTG​TGC​CTC​AGC​CTC​TTC​TC	ACT​GAT​GAG​AGG​GAG​CCC​AT	126	60

### Y-maze

The Y-maze test was conducted on the 35th day after MCAO/R surgery. In the Y-maze test, a maze constructed with high-contrast colors was used, and Sprague-Dawley rats naturally prefer the black chamber. The experiment was conducted in a quiet testing room, where each animal was placed at the center of the maze, and its behavior was recorded continuously for 5 min. Between trials, the maze walls and floor were thoroughly cleaned with 75% ethanol to eliminate residual odors or cues that might influence subsequent performance, and each rat was tested only once. Arm entries and total distance were recorded from the videos, and the spontaneous alternation rate was calculated as: [(number of alternations)/(total arm entries −2)] × 100%.

### Novel object recognition (NOR)

The NOR test was conducted on the 32th day after MCAO/R surgery. In the NOR test, rats were first habituated to an empty box for 10 min on Day 1. On Day 2, they were allowed to freely explore two identical objects placed in the box for 10 min. The formal test was conducted on Day 3, during which one familiar object was replaced with a novel object, and rats were again allowed to explore freely for 10 min. To eliminate odor cues, the apparatus was cleaned with 75% ethanol after each trial. Exploratory behavior was strictly defined as nose-sniffing or paw-touching the objects, whereas climbing or sitting on them was not counted. The recognition rate was calculated as the time spent exploring the novel object divided by the total time spent exploring both objects. In the present study, the objects were matched for height and color, and did not differ significantly in base area or surface texture, so as to eliminate the influence of low-level physical preferences. Behavioral scoring and video analyses were performed by investigators blinded to treatment allocation.

### Barnes maze

The Barnes maze test was conducted on the 42th day after MCAO/R surgery. The Barnes maze was used to evaluate spatial learning and memory retention after stroke. A dark escape box was positioned beneath one of the holes, and distal visual cues were placed at fixed locations around the maze. Each rat was placed in the center of the circular platform and allowed to explore freely. If the rat failed to locate the escape hole within 1 min, it was gently guided to the escape box and allowed to remain there for 15 s. Training was performed twice daily. After each session, the platform and escape box were cleaned with 75% ethanol to minimize olfactory cues. After 5 consecutive days of training, a probe test was conducted and escape latency (time to locate the escape hole) and total distance were recorded with a maximum cutoff of 120 s; latencies >120 s were assigned a value of 120 s. EthoVision XT software (17.0) was used to analyze the behavior. Data from the exploration period on day 6 (reflecting long-term memory retention) of the Barnes maze were primarily analyzed using a one-way ANOVA.

### Metagenomic analysis

Metagenomic sequencing was conducted in sham rats (n = 4), MCAO rats (n = 4) and MCAO + GV-971 rats (n = 5). Metagenomic sequencing was performed by Shanghai Meiji Biotechnology Co., Ltd. The procedures were as follows. DNA extraction: Microbial DNA was extracted using the Fast Pure Stool DNA Isolation Kit (MJYH, Shanghai, China). DNA concentration and purity were evaluated using a microvolume spectrophotometer (Nano Drop 2000; Thermo Fisher Scientific), and integrity was evaluated by 1% agarose gel electrophoresis. DNA was sheared to an average fragment size of ∼350 bp using a Covaris M220 (Gene Company Limited, China) for library preparation. Library preparation: Paired-end libraries were constructed using the NEXTFLEX Rapid DNA-Seq Kit (Bioo Scientific, United States), including adaptor ligation, magnetic bead–based size selection to remove self-ligated products, PCR amplification for library enrichment, and bead-based cleanup to obtain qualified libraries. Cluster generation and sequencing: Libraries were subjected to bridge amplification on an Illumina NovaSeq X Plus system (Illumina, United States) to generate clusters, and sequencing was performed using sequencing-by-synthesis to produce paired-end metagenomic reads.

Bioinformatic analysis of the sequencing data was performed by the Clinical translation testing platform, Shandong Laboratory of Yantai Drug Discovery. Raw paired-end metagenomic sequencing data were processed using the Meteor workflow. Briefly, meteor fastq was first used to index and organize sample-level FASTQ files. Reads were then mapped to the rat gut reference catalog (rn_5_9_gut) using meteor mapping, followed by taxonomic and functional profiling with meteor profile -n coverage (gene-length normalization enabled by -n coverage). Finally, sample-level outputs were merged using meteor merge to generate unified matrices for downstream analyses. According to the official Meteor documentation, the resulting outputs include MSP (Metagenomic Species Pangenomes) abundance and taxonomy tables, together with functional abundance matrices (e.g., KEGG KO and KEGG Module). Before downstream analyses, consistency checks were performed across MSP abundance tables, taxonomic annotation files, and sample metadata, and only samples shared by all data sources were retained. Taxonomy was harmonized into seven ranks (Kingdom, Phylum, Class, Order, Family, Genus, Species). To reduce noise from sparse features, taxa were filtered using dual criteria (mean relative abundance >0.01% and prevalence in at least 10% of samples) for community structure and differential analyses, while unfiltered abundance data were retained for alpha-diversity estimation.

Alpha diversity was assessed on unfiltered abundance data after rarefaction to a common sequencing depth. Diversity metrics included Shannon index, and Simpson dominance. Rarefaction to a common sequencing depth was used to control for uneven sequencing effort, as it was currently the only approach demonstrated to consistently control for sequencing effort across both alpha- and beta-diversity metrics. Sequencing depth did not differ significantly across groups (Kruskal–Wallis *P* = 0.585). To confirm robustness, all alpha diversity indices were recomputed on unrarefied raw count data using the same statistical comparison framework. Group comparisons were performed using a nonparametric framework (Wilcoxon rank-sum test for two-group comparisons and Dunn’s test for multi-group comparisons), with Benjamini-Hochberg correction for multiple testing. Beta diversity was evaluated at the MSP (species) level. Bray-Curtis distance (relative-abundance based) were calculated and visualized by PCoA. Statistical inference was performed using PERMANOVA (999 permutations), with additional pairwise PERMANOVA and multiple-testing correction for multi-group settings. Homogeneity of dispersion was assessed using betadisper, and ANOSIM (9,999 permutations) was used as a complementary rank-based test. Taxonomic composition was summarized across Phylum and Species levels. Dominant taxa were ranked by mean relative abundance within each rank, and low-abundance taxa were grouped as “Others”. Results were presented at both sample level and group-mean level to jointly describe individual heterogeneity and group-wise compositional patterns. Differential abundance analysis was performed only for pairwise comparisons, across taxonomic levels from Phylum to Species. Differential abundance analyses used LEfSe (Linear Discriminant Analysis Effect Size) to identify differentially abundant taxa through a dual-criteria approach: Kruskal-Wallis and Wilcoxon tests with raw *P*-value threshold of 0.05, combined with LDA effect size threshold of |LDA| > 2. This method identifies taxa that are both statistically significant and have substantial effect sizes, providing robust identification of biomarkers differentiating the compared groups. It should be noted that LEfSe did not apply multiple-testing correction by design; false-positive control relied on the combination of significance testing (Kruskal–Wallis and pairwise Wilcoxon tests) and an LDA effect-size threshold (|LDA score| > 2) rather than on formal FDR control. The absence of FDR correction was a recognized limitation that could inflate false-positive rates when many taxa were tested simultaneously, and LEfSe biomarker results therefore had to be interpreted with appropriate caution. Functional differences were assessed using Meteor-derived KEGG KO and KEGG Module abundance matrices. After sample matching, MaAsLin2 was used to model between-group functional differences (with normalization and log-transformation settings), and Wilcoxon rank-sum testing was used as complementary statistical evidence. Multiple testing was controlled using Benjamini–Hochberg correction. Major functional differences were interpreted jointly by statistical significance and effect size.

### Transcriptomic analysis

Transcriptome sequencing was conducted in 6 sham rats, 6 MCAO rats and 6 MCAO + GV-971 rats. 24 h after surgery, rats were transcardially perfused with pre-chilled saline solution, and their brain tissues were rapidly collected. Cortical tissue was collected at coordinates ML −2 mm, AP +0.5 mm, and DV −1 mm as cylindrical punches (1 mm in diameter, 1.5 mm in thickness), transferred to RNase-free tubes, and stored at −80 °C until processing. Transcriptome sequencing was performed by Shanghai Meiji Biotechnology Co., Ltd. RNA extraction: Tissue was lysed in QIAzol Lysis Reagent (cat. no. 79306, Qiagen), and total RNA was extracted in accordance with the manufacturer’s instructions. RNA concentration, purity, and integrity were evaluated using a microvolume spectrophotometer (Nano Drop 2000; Thermo Fisher Scientific) and agarose gel electrophoresis. Library preparation: Depending on the RNA input amount, libraries were prepared using the Illumina Stranded mRNA Prep Ligation workflow. Briefly, poly (A)+ mRNA was enriched using oligo (dT) beads, fragmented, and reverse-transcribed to generate cDNA, followed by adaptor ligation to complete library construction. Sequencing: After quality control, libraries were converted to single-stranded DNA, uncyclized molecules were removed, and DNA nanoballs were generated. Sequencing was then performed on a DNBSEQ-T7 platform to obtain transcriptomic reads. Bioinformatic analysis of the sequencing data was performed by the Clinical Translation Testing Platform of the Shandong Provincial Laboratory for New Drug Discovery and Development (Yantai, China). RNA-seq data were processed using the nf-core/rnaseq pipeline (v3.14.0). Raw reads were quality-checked with FastQC, followed by adaptor trimming and low-quality read filtering using Trim Galore. Reads were aligned to the *Rattus norvegicus* reference genome (mRatBN7.2) using STAR, and transcript abundance was quantified using Salmon to generate a gene-level count matrix. Differential expression and functional enrichment analyses were performed using the nf-core/differentialabundance pipeline (v1.5.0). Differential expression was tested in DESeq2 using the gene-count matrix, with a minimum abundance threshold of 20 and a minimum proportion threshold of 0.5. *P* values were adjusted using the Benjamini–Hochberg method, and differentially expressed genes were defined as those with an adjusted *P* value (padj) < 0.05. Functional enrichment was evaluated using gProfiler2 to test for the enrichment of KEGG pathways and transcription factors via hypergeometric tests with Benjamini–Hochberg correction. KEGG enrichment identified pathways overrepresented among differentially expressed genes, whereas transcription-factor enrichment identified putative upstream regulators.

### Determination of microbiota dependency

The animals were randomly assigned to one of five groups: Sham, MCAO/R, GV-971, ABX, and ABX + GV-971. For antibiotic treatment, four broad-spectrum antibiotics metronidazole (1 mg/mL, 443-48-1, Sangon Biotech), ampicillin (1 mg/mL, 69-52-3, Sangon Biotech), vancomycin (0.5 mg/mL, 1,404-93-9, Sangon Biotech), neomycin sulfate (1 mg/mL, 1,405-10-3, Sangon Biotech) were dissolved in the drinking water for a week (Zhang S. et al., 2024). The antibiotic solution was replaced every 2 days. Antibiotic treatment was continued throughout the entire experimental period. After 1 week of antibiotic administration, the animals received daily intragastric gavage of either GV-971 (0.3 mg/kg) or an equivalent volume of vehicle for three consecutive days prior to surgery. Subsequently, MCAO/R surgery was performed. 24 h after surgery, the rats were euthanized, and their brain tissues were immediately collected. Brain tissues were stained with 1% TTC to evaluate the volume of cerebral ischemic infarction.

### Fecal microbiota transplantation (FMT)

Fecal samples of all the groups were collected 24 h after surgery. Four broad-spectrum antibiotics metronidazole (1 mg/mL; CAS 443-48-1, Sangon Biotech), ampicillin (1 mg/mL; CAS 69-52-3, Sangon Biotech), vancomycin (0.5 mg/mL; CAS 1404-93-9, Sangon Biotech), and neomycin sulfate (1 mg/mL; CAS 1405-10-3, Sangon Biotech) were dissolved in drinking water to deplete the gut microbiota. The antibiotic solution was replaced every 2 days and continued for 7 days. All the animals were then switched to antibiotic-free drinking water before fecal microbiota transplantation (FMT). The pooled donors of the corresponding group were collected, donor feces were collected from the Sham, MCAO/R, and GV-971 (0.3 mg/kg) groups. Frozen samples were thawed in a 37 °C water bath and resuspended in sterile 1×PBS (100 mg feces per 1 mL). The suspension was mixed and vortexed for 5 min, centrifuged at 800 *g* for 5 min, and the supernatant was collected. The supernatant was immediately transferred into an isolator under sterile pass-through conditions and used for FMT. Each recipient rat received 1 mL of the corresponding fecal supernatant once daily by intragastric gavage. MCAO surgery was performed after 3 days of pre-treatment with the fecal supernatant, and FMT was continued on the day of surgery and the following day. Brain tissues were collected 24 h after surgery for 1% TTC staining–based infarct analysis.

### Metabolomics analysis

Metabolomics analysis was conducted in 5 sham rats, 5 MCAO rats and 5 MCAO + GV-971 rats. Fecal samples were collected from rats 24 h after reperfusion and stored at −80 °C. Sample preparation was carried out by Metabo-Profile Biotechnology (Shanghai) Co., Ltd. Metabolomic profiling was performed using the Q500 kit (Metabo-Profile, Shanghai, China). Briefly, samples stored in Eppendorf Safelock microcentrifuge tubes were mixed with 10 precooled zirconia beads and 20 µL of deionized water. The samples were homogenized for 3 min, after which 150 µL of methanol containing internal standards was added for metabolite extraction. The samples were homogenized again for 3 min and then centrifuged at 18,000 × g for 20 min. The resulting supernatants were transferred to 96-well plates. Subsequent operations were conducted on a Biomek 4,000 workstation (Beckman Coulter, Inc., Brea, CA, United States). To each well, 20 µL of freshly prepared derivatization reagent was added. After sealing, the plates were incubated at 30 °C for 60 min to allow derivatization. Following derivatization, the samples were evaporated for 2 h, and the residues were reconstituted in 330 µL of ice-cold 50% methanol. The plates were then kept at −20 °C for 20 min and centrifuged at 4,000 × g for 30 min at 4 °C. An aliquot of 135 µL of the supernatant was transferred to a new 96-well plate, each well of which had been preloaded with 10 µL of internal standard. A series of diluted derivatized standards were added to the left-side wells. Finally, the plates were sealed for liquid chromatography–mass spectrometry (LC–MS) analysis.

All reference standards were purchased from Sigma-Aldrich (St. Louis, MO, United States), Steraloids Inc. (Newport, RI, United States), and TRC Chemicals (Toronto, ON, Canada). Each standard was precisely weighed and individually dissolved in water, methanol, sodium hydroxide solution, or hydrochloric acid to prepare single-component stock solutions at a concentration of 5.0 mg/mL. These stock solutions were then mixed in appropriate proportions to generate the calibration stock mixture.

In this study, quantitative analysis of all target metabolites was performed by Metabo-Profile Biotechnology (Shanghai) Co., Ltd. using an ultra-performance liquid chromatography–tandem mass spectrometry (UPLC–MS/MS) system (ACQUITY UPLC-Xevo TQ-S, Waters Corp., Milford, MA, United States of America). Data processing, including integration, interpretation, and visualization, was conducted using proprietary software developed by Metabo-Profile.

For metabolomic data analysis, two broad categories of statistical approaches were employed (An et al., 2025): multivariate analyses, including principal component analysis (PCA), partial least-squares discriminant analysis (PLS-DA), orthogonal partial least-squares discriminant analysis (OPLS-DA), random forest, and support vector machine learning; and (GBD 2019 Stroke Collaborators, 2021) univariate analyses, comprising Student’s t-test, Mann–Whitney Wilcoxon test, analysis of variance (ANOVA), and correlation analysis.

### Statistical analysis

Multiple comparisons among groups were conducted using one-way analysis of variance (ANOVA) followed by Dunnett’s test. Shapiro–Wilk and Levene’s tests were used to justify the choice between parametric and Kruskal–Wallis non-parametric analyses. Statistical analyses were performed in GraphPad Prism (v9.5.1). Data were presented as mean ± sem, and *P* < 0.05 was considered statistically significant. The sample size was based on previous studies with similar experimental paradigms (Huang et al., 2025; Luo et al., 2025).

## Results

### GV-971 treatment conferred neuroprotection in a rat model of ischemic stroke

**FIGURE 1:**
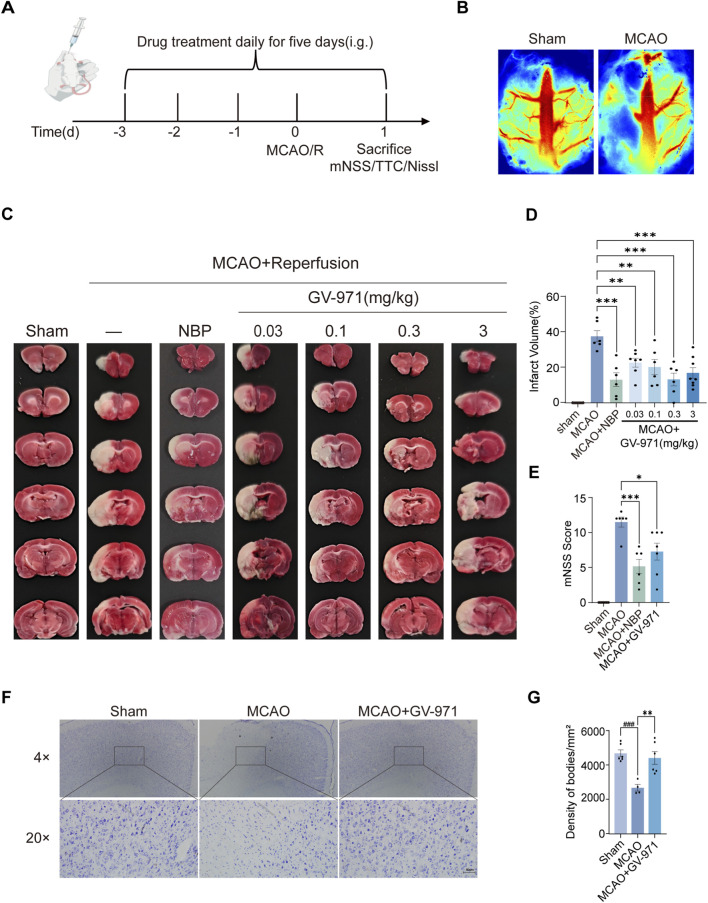
GV-971 treatment conferred neuroprotection in a rat model of ischemic stroke (IS). **(A)** Rat MCAO Modeling Experimental Flow Chart. **(B)** Laser Speckle Flow Imaging Pseudocolor Map. **(C)** Representative schematic diagram of TTC staining in brain tissue (n = 6–10). **(D)** Quantification of cerebral infarct volume (n = 6–10). **(E)** Quantification of Neurological impairment scores (mNSS) (n = 6–10). **(F,G)** Nissl staining and quantification in the ipsilateral ischemic penumbra (n = 4–6). Scale bar = 50 μm. Data were presented as mean ± sem. Statistical method: one-way ANOVA. *P < 0.05, **P < 0.01, ***P < 0.001 versus MCAO; ^###^ *P <* 0.001 versus Sham.

First, Rats underwent middle cerebral artery occlusion/reperfusion (MCAO/R). Laser speckle contrast imaging performed 24 h after surgery showed cerebral perfusion status in the ischemic hemisphere, with significantly reduced blood flow compared to the contralateral hemisphere (Figures 1A,B). To evaluate neuroprotection, MCAO/R rats were administered GV-971 by intragastric gavage at doses of 0.03, 0.1, 0.3, and 3 mg/kg. At 24 h after surgery, representative TTC staining revealed a pronounced increase in infarct volume in the MCAO/R gr,oup (Figure 1C). 3-n-Butylphthalide (NBP) is a neuroprotective agent approved for stroke treatment, with a reference dose of 12.5 mg/kg (Dai et al., 2023). Compared with the MCAO group, the NBP group and GV-971 treatment reduced infarct volume and improved neurological scores. The infarct volume of the NBP group decreased from 37.81% ± 2.391% to 13.19% ± 5.023% (*P =* 0.0006). GV-971 treatment reduced infarct volume with optimal efficacy observed at intermediate concentrations from 37.81% ± 2.391% to 22.807% ± 3.999%, 20.337% ± 5.336%, 13.30% ± 4.801% and 17.001% ± 4.447% (Figure 1D; *P* = 0.0032, 0.0084, 0.0005 and 0.0005, respectively). Compared with the MCAO group, neurological impairment score of NBP group and 0.3 mg/kg GV-971 treatment significantly decreased from 11.50 ± 0.54 to 5.167 ± 1.243 (*P =* 0.0005) and 7.29 ± 1.47 (Figure 1E; *P* = 0.0154), respectively. Notably, 0.3 mg/kg GV-971 treatment significantly attenuated neuronal loss in the ipsilateral cortex after ischemia-reperfusion. Neuronal density in the MCAO/R group decreased to 2,594 ± 306.8 cells/mm^2^ compared with the Sham group (*P =* 0.0002) and increased to 4,373 ± 499.7 cells/mm^2^ with GV-971 treatment compared with the MCAO group (*P =* 0.0074) (Figures 1F,G). RNA-seq was then performed on tissue from the ischemic hemisphere. Volcano plots revealed GV-971 mainly upregulated JPH4, which maintains ER-PM connections and calcium signaling, and upregulated Kcng4, which indirectly inhibits neuronal overexcitation. In addition, it also upregulated some genes related to antioxidant, vascular protection, and auxiliary neuronal survival, such as Notum, Nat8f3, Fzd9, and Hhatl (Supplementary Figure S1A). However, it should be emphasized that differential expression alone did not prove that these genes were directly involved in the neuroprotective phenotype. KEGG analysis highlighted enrichment in ECM-receptor interaction, focal adhesion, neuroactive ligand-receptor interaction, and the PI3K-Akt pathway, which was aimed at providing direction for subsequent mechanism research. These pathways converged on ECM remodeling, neural signaling, inflammation, and cell survival (Supplementary Figure S1B), aligning well with ischemic stroke pathogenesis and providing microscopic speculations for mechanistic dissection and target identification. In conclusion, GV-971 treatment reduced infarct volume and improved neurological function after experimental stroke, and transcriptomic profiling of the ischemic hemisphere suggested that GV-971 treatment promoted post-stroke 640brain repair through multi-target regulation.

### GV-971 treatment conferred neuroprotection in the MCAO/R model by attenuating gliosis and limiting blood–brain barrier disruption

**FIGURE 2:**
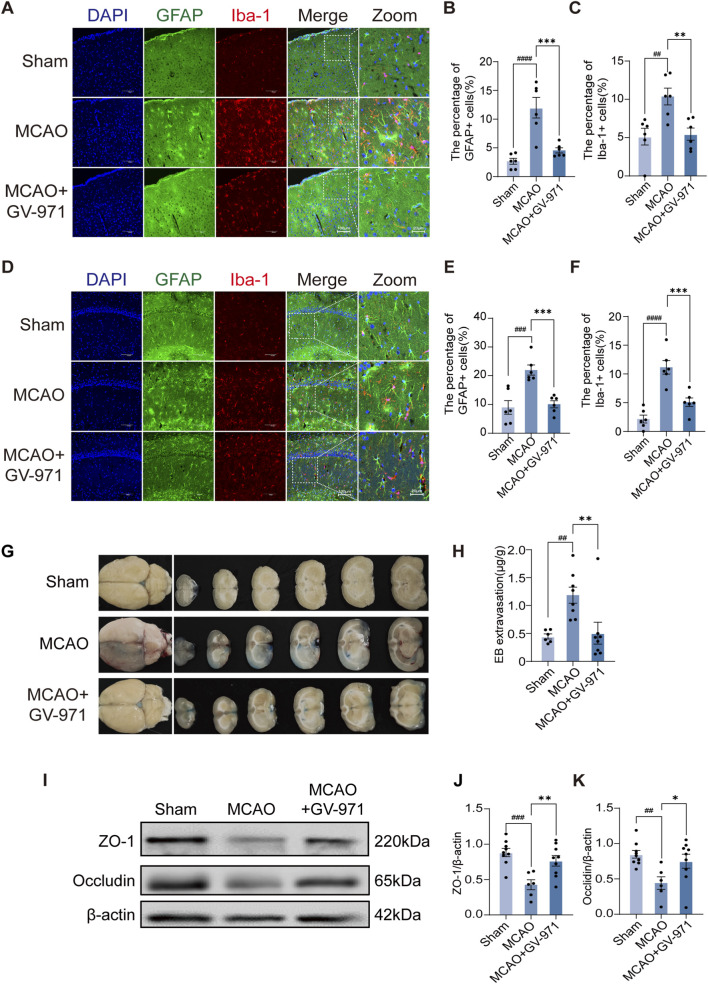
GV-971 treatment attenuated gliosis and preserved blood–brain barrier integrity. **(A–C)** Representative immunofluorescence images and quantification of GFAP and Iba-1 in the ipsilateral cerebral cortex (n = 6). Scale bar = 100 μm (Merge), Scale bar = 20 μm (Zoom). **(D–F)** Representative immunofluorescence images and quantification of GFAP and Iba-1 in the ipsilateral hippocampal CA1 region (n = 6). Scale bar = 100 μm (Merge), Scale bar = 20 μm (Zoom). **(G)** Representative Evans blue extravasation images in rat brain at 24 h after MCAO. **(H)** Quantification of Evans blue in ipsilateral brain tissue (n = 6–8). **(I–K)** Immunoblot analysis and quantification of ZO-1 and Occludin in the cerebral cortex (n = 6–9). Data were presented as mean ± sem. Statistical method: one-way ANOVA. *P < 0.05, **P < 0.01, ***P < 0.001 versus MCAO; ##*P <* 0.01, ###*P* < 0.001 versus Sham.

Ischemic stroke elicits a robust inflammatory response and gliosis in the central nervous system, during which astrocytes and microglia engage in dynamic crosstalk that shapes the inflammatory cascade and influences disease progression (Linnerbauer et al., 2020). During the acute phase of ischemia, reactive astrocytes and activated microglia release neurotrophic factors that can partially counteract inflammation-associated injury (Candelario-Jalil et al., 2022). Therefore, we employed immunofluorescence staining to assess astrocyte and microglial activation in the cortical penumbra and the ipsilateral hippocampal CA1 region, thereby evaluating the modulatory effect of GV-971 on gliosis. In the ipsilateral cortex, the MCAO group showed a marked increase in the percentage of activated astrocytes and microglia compared with the Sham group, from 2.657% ± 0.5140% and 5.126% ± 1.097% to 12.00% ± 1.792% (*P =* 0.0005) and 10.37% ± 1.101% (*P =* 0.0071), respectively. Consistent with this quantification, morphological analysis revealed that astrocytes in the MCAO group exhibited hypertrophic cell bodies and thickened, disorganized processes, while microglia transformed from a ramified to an amoeboid morphology with retracted processes and enlarged somata. GV-971 treatment significantly attenuated this glial activation, reducing the proportion of positive cells to 4.551% ± 0.4505% (*P =* 0.0005) and 5.455% ± 0.8049% (*P =* 0.0069), respectively, and restored the ramified morphology characteristic of the resting state (Figures 2A–C). A similar pattern was observed in the ipsilateral CA1 region, where GV-971 treatment significantly reduced the percentage of activated astrocytes and microglia to 10.10% ± 1.237% (*P =* 0.0007) and 5.081% ± 0.7440% (*P =* 0.0005), respectively, and reversed the pathological morphological changes (Figures 2D–F). The blood–brain barrier (BBB) maintains the CNS microenvironment required for normal neuronal function (Liebner et al., 2018) and is compromised after stroke (Villringer et al., 2017). To test whether GV-971 treatment preserves BBB integrity after MCAO/R, we quantified Evans blue extravasation as an index of BBB permeability. Compared with the Sham group, BBB permeability was markedly increased in the MCAO group. Notably, GV-971 treatment significantly attenuated this increase, reducing Evans blue extravasation to 0.5063 ± 0.1970 μg/g (*P =* 0.0087) (Figures 2G,H). Consistent with improved barrier integrity, immunoblotting showed that the tight-junction proteins ZO-1 and Occludin were decreased in the MCAO group and were restored by GV-971 treatment. Specifically, ZO-1 increased from 0.4274 ± 0.07022 to 0.7664 ± 0.07397 (*P =* 0.0061), and Occludin increased from 0.4409 ± 0.08943 to 0.7501 ± 0.09881 (*P =* 0.0365) (Figures 2I–K). These data indicated that GV-971 treatment attenuated MCAO/R-induced gliosis and mitigated concomitant BBB disruption.

### GV-971 treatment attenuated intestinal inflammation in rats subjected to ischemic stroke

**FIGURE 3:**
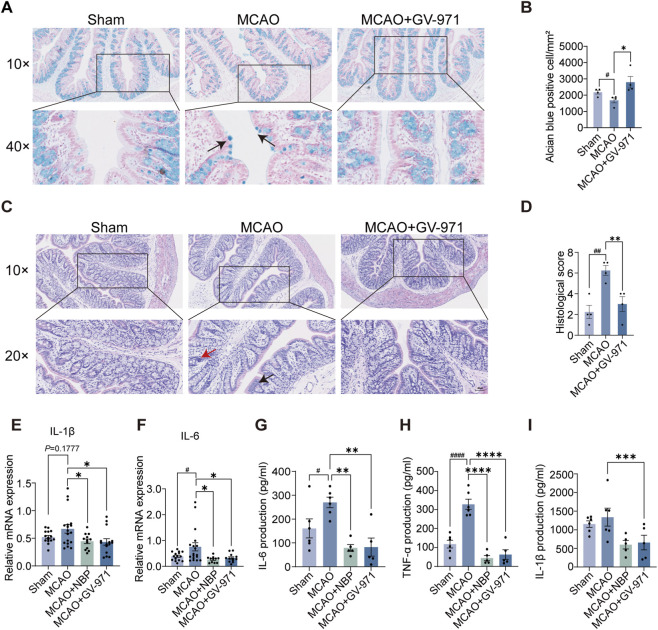
GV-971 treatment attenuated intestinal inflammation in rats subjected to ischemic stroke. **(A,B)** Representative Alcian blue–stained colon tissues and quantification. Black arrows indicate goblet cells released into the intestinal lumen, consistent with impaired goblet-cell positioning (n = 4–6). Scale bar = 2 0 μm. **(C,D)** Representative H&E-stained colon tissues and histopathological scoring. Black arrows indicated lymphocyte infiltration; red arrows indicated damaged crypt epithelium (n = 4–6). Scale bar = 50 μm. **(E)** Colon tissue IL-1β mRNA expression (n = 4–6). **(F)** Colon tissue IL-6 mRNA expression (n = 4–6). **(G)** Colon tissue levels of IL-6 measured by ELISA. (n = 5–6). **(H)** Colon tissue levels of TNF-α measured by ELISA. (n = 5–6) **(I)** Colon tissue levels of IL-1β measured by ELISA. (n = 5–6). Data were presented as mean ± sem. Statistical method: one-way ANOVA. *P < 0.05, **P < 0.01, ***P < 0.001, ****P < 0.0001 versus MCAO; #*P <* 0.05, ##*P <* 0.01, ####*P <* 0.0001 versus Sham.

Evidence supports bidirectional communication between the brain and gut following stroke. Disruption of the commensal microbiota after cerebral ischemia promotes the expansion of pathogenic taxa and the accumulation of inflammatory microbial metabolites, thereby amplifying intestinal inflammation (Sun et al., 2022)^.^ Cerebral ischemia also compromises the intestinal mucus layer produced by goblet cells, weakening barrier defense and promoting microbial invasion (Wang et al., 2024). Alcian blue staining evaluated mucus-layer integrity and showed disrupted mucosal architecture together with a marked loss of goblet cells after MCAO. GV-971 treatment strengthened the mucosal barrier and increased Alcian blue–positive goblet cells from 1,683 ± 156.4 to 2,794 ± 354.5 cells/mm^2^ (*P* = 0.0164) (Figures 3A,B). To assess whether GV-971 mitigates this peripheral inflammatory response, we performed H&E staining on colon tissue. Consistently, H&E staining showed that GV-971 treatment attenuated goblet-cell depletion, crypt abscess formation and inflammatory cell infiltration, reducing the histopathology score from 6.250 ± 0.4787 to 3.00 ± 0.7071 (*P* = 0.0083) (Figures 3C,D). We also performed RT–qPCR on colon tissue to quantify the mRNA expression of key inflammatory mediators. Compared with the MCAO group, GV-971 treatment significantly downregulated the mRNA levels of IL-1β and IL-6. Specifically, IL-1β levels decreased from 0.6701 ± 0.07637 (MCAO) to 0.4327 ± 0.05862 (GV-971) (*P* = 0.0194), and IL-6 levels decreased from 0.7556 ± 0.1591 (MCAO) to 0.3225 ± 0.04708 (GV-971) (*P* = 0.0327) (Figures 3E,F). At the protein level, ELISA results demonstrated that treatment with GV-971 significantly reduced the expression levels of pro-inflammatory cytokines in colonic tissues compared with the MCAO group. Specifically, interleukin-6 (IL-6) levels decreased from 270.2 ± 21.99 pg/mL protein to 82.10 ± 38.01 pg/mL protein (*P* = 0.0014). Similarly, tumor necrosis factor-alpha (TNF-α) expression was markedly suppressed from 327.9 ± 26.75 pg/mL to 62.59 ± 25.51 pg/mg (*P* < 0.0001), and interleukin-1 beta (IL-1β) levels dropped from 219.9 ± 19.95 pg/mg to 47.77 ± 7.945 pg/mL (*P* = 0.0003) (Figures 3G–I). Overall, these results indicated that GV-971 treatment limited the intestinal inflammatory response exacerbated by ischemia-reperfusion injury.

### GV-971-mediated protection against ischemic stroke depended on the gut microbiota

**FIGURE 4:**
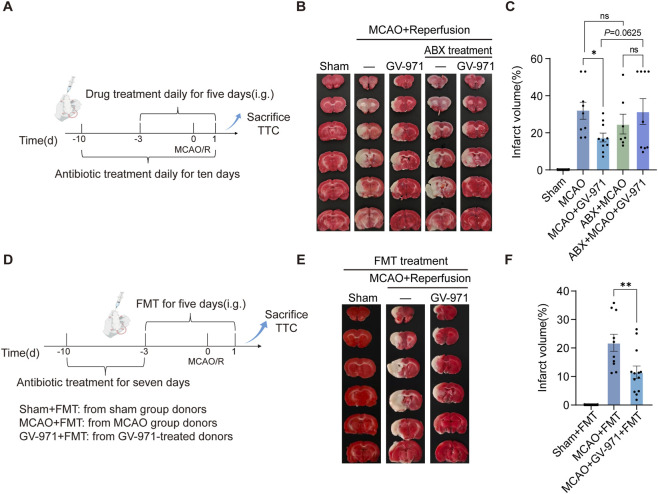
GV-971-mediated protection against ischemic stroke depended on the gut microbiota. **(A)** Experimental schematic for efficacy assessment after antibiotic treatment. **(B)** Representative TTC-stained brain tissues. **(C)** Quantification of cerebral infarct volume (n = 7–10). **(D)** Experimental schematic for fecal microbiota transplantation (FMT). **(E)** Representative schematic diagram of TTC staining in brain tissue. **(F)** Quantification of cerebral infarct volume (n = 10–13). Data were presented as mean ± sem. Statistical method: one-way ANOVA. *P < 0.05, **P < 0.01versus MCAO.

GV-971 is a gut microbiota modulator. To test whether its therapeutic efficacy depended on the microbiota, animals underwent antibiotic-mediated microbiota depletion before MCAO/R. GV-971 treatment reduced infarct volume in microbiota-intact rats, decreasing infarct volume from 31.91% ± 4.612% in the MCAO group to 17.44% ± 2.380% (*P =* 0.0327). In contrast, after antibiotic treatment, the GV-971 group failed to further reduce infarct volume compared with the antibiotic-treated MCAO group (24.71% ± 5.328% versus 31.50% ± 6.973%; *P =* 0.9990). There was no significant difference between the GV-971 group and the ABX + GV-971 group (*P* = 0.3315) (Figures 4A–C). These results indicated that protection against ischemic injury depended on the gut microbiota. To further examine the contribution of the microbiota to stroke outcomes, antibiotic-treated recipient rats underwent fecal microbiota transplantation (FMT). MCAO/R recipients received FMT from GV-971-treatedrats, whereas Sham and MCAO/R control recipients received the corresponding donor microbiota. The GV-971+FMT group reduced infarct volume compared with the MCAO group, decreasing infarct size from 21.84% ± 3.022% to 11.53% ± 2.160% (*P =* 0.0015) (Figures 4D–F). In brief, these data supported a causal role for the gut microbiota in modulating ischemic stroke severity and indicated that GV-971 acted, at least in part, through microbiota-dependent mechanisms.

### GV-971 treatment modulated the gut microbiota in MCAO/R rats

**FIGURE 5:**
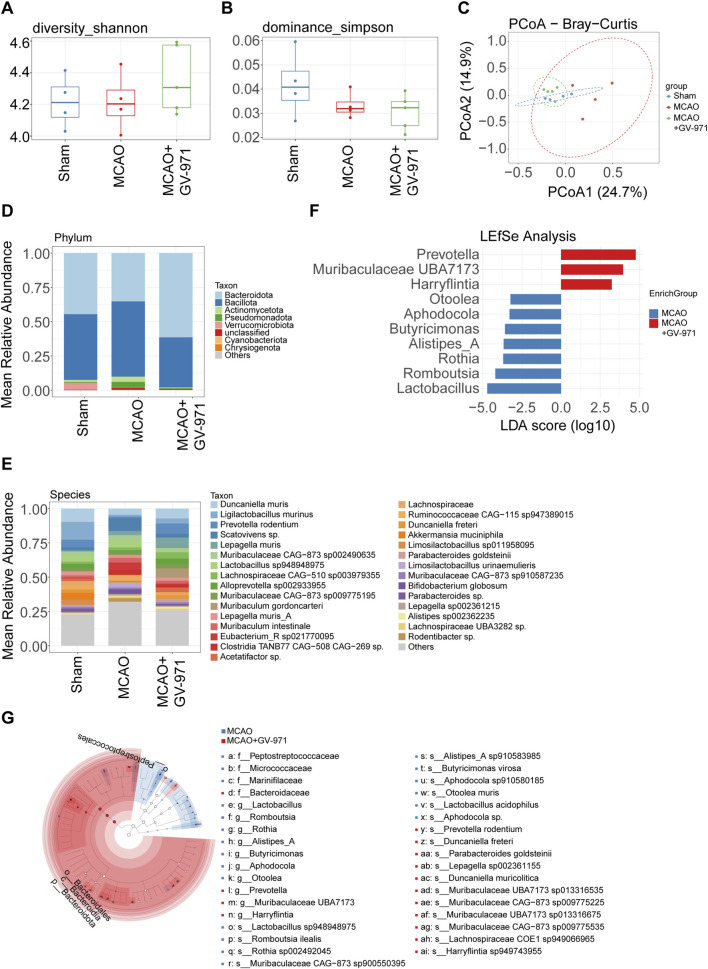
GV-971 treatment modulated the gut microbiota in MCAO/R rats. **(A)** Alpha diversity evaluated by the Shannon index. **(B)** Alpha diversity evaluated by the Simpson index. **(C)** Principal coordinate analysis (PCoA) based on Bray–Curtis distances. **(D)** Relative abundance at the phylum level. **(E)** Relative abundance at the species level. **(F)** Genus LDA score plot. **(G)** LEfSe analysis (n = 4–5).

The gut microbiota is a key regulator of ischemic stroke pathogenesis (Zhang J. et al., 2024)^.^ Stroke onset and progression are frequently accompanied by microbial dysbiosis and translocation, typically characterized by an expansion of potentially pathogenic taxa and a loss of beneficial commensals (Wang et al., 2023). We therefore investigated whether GV-971 treatment reshaped the gut microbiota and its functional potential after middle cerebral artery occlusion/reperfusion (MCAO/R). Shotgun metagenomic sequencing of fecal samples was performed to profile community composition and inferred metabolic capacity after MCAO/R and GV-971 treatment. Although GV-971 treatment did not significantly alter global alpha diversity metrics, as evidenced by comparable Shannon and Simpson indices across Sham, MCAO, and GV-971 groups (Figures 5A,B), it induced a profound restructuring of gut microbiota composition at the beta-diversity level. Principal coordinate analysis (PCoA) based on Bray-Curtis dissimilarity revealed clear separation of the GV-971 group from both Sham and MCAO groups along PCoA1 (24.7%) and PCoA2 (14.9%) (Figure 5C). This indicates that GV-971 drives a distinct microbial community configuration rather than merely restoring richness or evenness. Visual inspection of taxonomic profiles confirmed a GV-971-induced structural shift. At the phylum level (Figure 5D), while Bacteroidota and Bacillota remained dominant across all groups, MCAO surgery induced a distinct dysbiosis characterized by decreased Bacteroidota and enriched Bacillota compared to Sham group. Notably, GV-971 treatment reversed these aberrations, restoring Bacteroidota abundance while suppressing Bacillota. These macro-level changes were driven by specific fluctuations at the species level (Figure 5E). GV-971 significantly enriched beneficial taxa, including *Duncaniella muris*, *Ligilactobacillus murinus*, *Prevotella rodentium*, and *Akkermansia muciniphila*, which are known for their roles in short-chain fatty acid (SCFA) production and gut barrier maintenance (Zhu et al., 2024)^.^ Short-chain fatty acid changes in feces are presented in the supplementary data. Fecal metabolomics showed that GV-971 administration increased all three major SCFAs compared with the MCAO group: acetic acid from 18,404 ± 2,728 to 25,389 ± 1,421 nmol/g (*P* = 0.0515), butyric acid from 3,274 ± 959.4 to 4,246 ± 690.4 nmol/g (*P* = 0.6375), and propionic acid from 3,973 ± 718.8 to 4,902 ± 326.6 nmol/g (*P* = 0.3894). Although not statistically significant, the consistent upward trends suggest a potential role for GV-971 in promoting SCFA production (Supplementary Figure S4). Collectively, this restoration of key taxa likely limits bacterial translocation and systemic inflammation, thereby attenuating neuroinflammatory injury.

This structural remodeling was further corroborated by LEfSe analysis, which identified specific taxonomic biomarkers associated with each condition (Figure 5F). Specifically, the GV-971 group was significantly enriched in Prevotella, known for its role in polysaccharide fermentation and anti-inflammatory short-chain fatty acid production (Filippis et al., 2016); Muribaculaceae UBA7173, linked to synergistic fructan-driven acetate production and inflammation amelioration (Takahashi et al., 2025); and Harryflintia, an acetate-producing, spore-forming anaerobe belonging to *Clostridium* cluster IV (Petzoldt et al., 2016). In contrast, the MCAO group exhibited enrichment of potentially pro-inflammatory or dysbiosis-associated taxa, including Rothia, whose expansion in this context may signify ischemia-driven ecological imbalance (Kisilevsky et al., 2021); Otoolea, recently implicated in radiation-induced enteritis (Zhong et al., 2025); Romboutsia and *Lactobacillus*, often elevated under stress or inflammation; and Alistipes_A and Butyricimonas, whose context-dependent roles may shift toward pathogenicity in diseased states (Parker et al., 2020; Chen Y. Z. et al., 2024). Notably, the suppression of Rothia, Otoolea, and other MCAO-enriched taxa by GV-971 suggests a targeted reversal of ischemia-induced dysbiosis. Phylogenetic cladogram analysis further confirmed these shifts within evolutionary lineages, highlighting GV-971’s preferential promotion of Bacteroidales-derived beneficial clades over Bacillota-associated taxa prevalent in MCAO animals (Figure 5G). Collectively, these findings demonstrate that GV-971 exerts its therapeutic effect not through broad diversification of the microbiome, but via precise qualitative reprogramming of microbial community structure. By enriching symbiotic, metabolically active taxa while suppressing those linked to post-ischemic pathology, GV-971 potentially restores gut-brain axis homeostasis.

To characterize the metabolic impact of GV-971 treatment following MCAO, we compared enzyme-level and pathway-level functional profiles between the 0.3 mg/kg GV-971-treated group and the MCAO group. At the enzyme level (Supplementary Figure S2A), we analyzed the mean relative abundance of KEGG orthologs (KOs) and highlighted the top 30 KOs exhibiting the most pronounced shifts. The majority exhibited significantly higher abundance in the GV-971 group, particularly those involved in central carbon metabolism (e.g., K00602, K00174, K00175), amino acid transamination (K00812, K00831), nucleotide interconversion (K00941, K00942), and redox cofactor regeneration (K00344, K00262). The right panel, plotting the difference in means (calculated as MCAO minus GV-971), shows predominantly negative values with confidence intervals not crossing zero, confirming statistically robust upregulation under GV-971 treatment. This indicates that GV-971 has the potential to enhance the enzymatic abilities of energy production, biosynthesis, and antioxidant defense after ischemic.

At the pathway level (Supplementary Figure S2B), we assessed MetaCyc pathways identifiable by their unique prefixes (M, MF, MGB) and presented the 30 pathways with the most substantial variations. These data reveal a coordinated shift toward anabolic and protective metabolism in the GV-971 group. Biosynthetic pathways including lysine (M00527, M00526), histidine (M00026, M00045), cysteine (M00021), and UDP-sugar (M00549, M00554) metabolism were markedly elevated, alongside gluconeogenesis (M00003) and NAD biosynthesis (M00115), indicating that GV-971 has the potential for tissue repair and redox homeostasis. Conversely, fermentative or degradative routes such as ethanol production via formate pathway (MF0001) and galactose degradation (M00632, MF0056) were suppressed or unchanged. The difference-in-means plot (right panel) again confirms widespread negative shifts (i.e., higher abundance in GV-971). Collectively, these data demonstrate that GV-971 reprograms post-MCAO metabolism away from catabolic stress responses and toward regenerative, energetically supportive, and antioxidative states, thereby providing a theoretical basis for its neuroprotective efficacy.

### GV-971 treatment improved motor and cognitive impairment in rats subjected to MCAO/R

**FIGURE 6:**
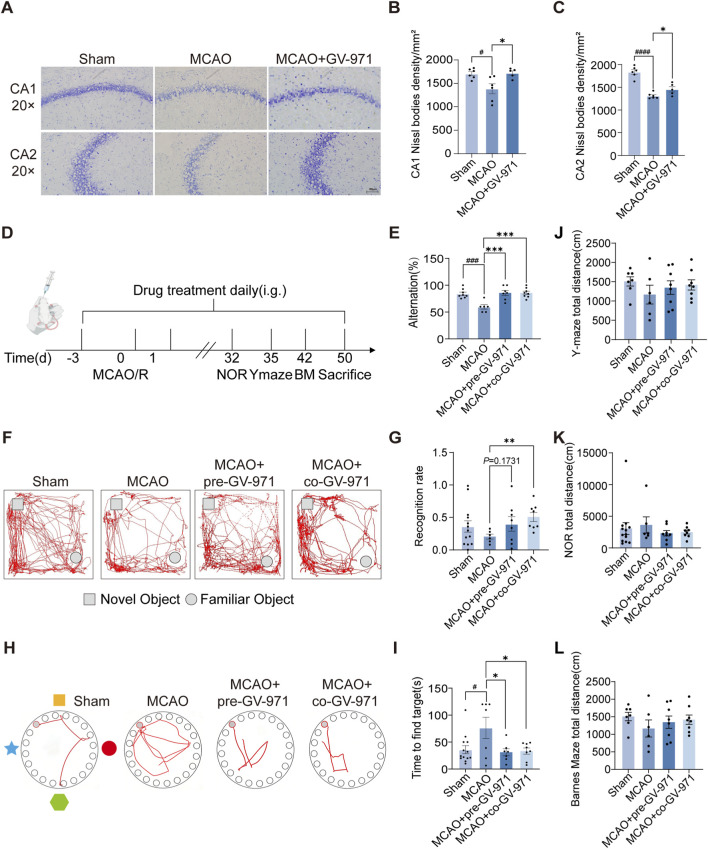
GV-971 treatment improved motor and cognitive impairment in rats subjected to MCAO/R. **(A)** Representative Nissl staining of hippocampal CA1 and CA2 regions at 24 h after MCAO/R (n = 5–6). **(B)** Quantification of Nissl bodies in CA1. **(C)** Quantification of Nissl bodies in CA2 (n = 5–6). **(D)** Schematic of the long-term behavioral testing paradigm. **(E)** Y-maze performance on day 35 (n = 6–8). **(F)** Representative trajectories and quantification for novel object recognition (NOR) on day 32. **(G)** NOR recognition index (n = 6–13). **(H,I)** Representative trajectories and quantification analysis for the Barnes maze test (n = 7–13). **(J)** Y-maze total distance. (n = 6–8). **(K)** NOR total distance. (n = 6–13). **(L)** Barnes Maze total distance. (n = 7–13). Data were presented as mean ± sem. Statistical method: one-way ANOVA. *P < 0.05, **P < 0.01, ***P < 0.001 versus MCAO; #*P <* 0.05, ###*P <* 0.001, ####*P <* 0.0001 versus Sham. pre-GV-971 denoted pre-administration, co-GV-971 denoted administration during reperfusion.

Evidence indicates that MCAO rats develop dementia-like phenotypes and are therefore widely used as a model of vascular cognitive impairment (Yang et al., 2006)^.^ We further evaluated whether GV-971 treatment improved cognitive function after MCAO/R in rats. Nissl staining showed that, compared with Sham, the 24 h MCAO group exhibited a marked loss of neuronal Nissl bodies in the hippocampal CA1 and CA2 regions, whereas GV-971 treatment preserved neuronal density and cytoarchitecture. Compared with MCAO/R, neuronal Nissl body density increased from 1,385.33 ± 114.4 to 1,720.6 ± 123.7 cells/mm^2^ in CA1 (*P =* 0.0134) and from 1,335 ± 57.16 to 1,720.6 ± 59.05 cells/mm^2^ in CA2 (*P =* 0.0362) (Figures 6A–C). To further test cognitive function, animals underwent the Y-maze, novel object recognition and Barnes maze assays (Figure 6D). In the Y-maze, GV-971 administered either before MCAO/R (pre-treatment) or during the perioperative period (co-treatment) increased spontaneous alternation rate compared with MCAO/R, from 60.95% ± 4.91% to 85.60% ± 6.32% (*P =* 0.0002) and 85.64% ± 5.027% (*P =* 0.0002), respectively (Figure 6E). In the novel object recognition assay, MCAO/R reduced the recognition index compared with Sham, whereas GV-971 treatment increased new-object exploration from 0.2039 ± 0.03752 to 0.3991 ± 0.1122 (pre-treatment) and 0.5066 ± 0.06982 (co-treatment) (*P* = 0.1731, *P =* 0.0047), respectively (Figures 6F,G). In the Barnes maze, MCAO/R prolonged escape latency, and GV-971 treatment reduced the time required to locate the target hole compared with the MCAO group from 76.45 ± 17.41 s to 31.03 ± 20.75 s (*P* = 0.0201) and 33.37 ± 19.30 s (*P* = 0.0225) for pre- and co-treatment, respectively (Figures 6H,I). Behavioral assessments revealed that no significant differences were observed among groups in either the Y-maze total distance traveled or the NOR total distance (*P* > 0.05), nor in the total distance traveled in the Barnes Maze test (*P* > 0.05), indicating that the cognitive improvements observed in this study were not attributable to intergroup differences in locomotor activity (Figures 6J–L). Overall, histological and behavioral analyses showed that MCAO/R induced hippocampal neuronal loss and cognitive impairment, and that GV-971 treatment improved post-stroke learning and memory performance.

## Discussion

This study demonstrates that sodium oligomannate (GV-971) exerts robust neuroprotective effects in a rat model of ischemic stroke (MCAO/R). Specifically, GV-971 treatment significantly reduced cerebral infarct volume, attenuated glial activation, preserved blood–brain barrier (BBB) integrity, and improved both motor and cognitive functions. Importantly, we demonstrated that these therapeutic benefits are mediated through the remodeling of gut microbiota and the suppression of peripheral inflammation, indicating that GV-971 has the potential to restore gut brain axis homeostasis.

Ischemic stroke triggers a pathological cascade involving gliosis, BBB disruption, and neuronal death. In the acute phase, activated microglia and astrocytes drive the inflammatory response, which can compromise BBB tight junctions and amplify secondary injury (Zhang et al., 2018; Lu et al., 2026; Xu et al., 2026). Our findings show that GV-971 effectively interrupted this cascade: immunofluorescence analysis revealed a significant reduction in the activation of astrocytes and microglia in both the cortex and hippocampus. This suppression of glial reactivity was associated with the restoration of tight-junction proteins and reduced Evans blue extravasation. Furthermore, transcriptomic profiling of the ischemic hemisphere indicated that GV-971 treatment modulated ECM remodeling, neural signaling, inflammation, and cell survival (Supplementary Figure S2B), upregulated JPH4, Kcng4 and some genes related to antioxidant, vascular protection, and auxiliary neuronal survival, such as Notum, Nat8f3, Fzd9, and Hhatl. By dampening central glial activation and modulating inflammatory signaling, GV-971 likely interrupted the cycle of secondary injury, thereby preserving the CNS microenvironment. Although these findings highlight several candidate molecules, independent orthogonal assays are required to corroborate their functional relevance.

Despite promising preclinical data, most anti-inflammatory therapies have failed in clinical trials due to the complexity of ischemic cascades, the difficulty of crossing the compromised BBB, and narrow therapeutic windows. GV-971 offers a distinct advantage by addressing these specific translational barriers. First, rather than targeting a single pathway, GV-971 acts as a multi-target modulator, simultaneously reshaping gut microbiota, suppressing peripheral cytokines, and inhibiting central glial activation. Second, by leveraging the gut–brain axis, it circumvents the critical challenge of direct BBB penetration; our data confirm that peripheral modulation of colonic inflammation effectively correlates with reduced gliosis, suggesting an indirect yet potent mechanism to protect the brain. Finally, given its efficacy in improving long-term cognitive outcomes and its approved indication for Alzheimer’s disease, GV-971 may offer a wider therapeutic window than acute neuroprotectants, addressing the persistent inflammatory phase that extends well beyond the initial injury.

The causal role of the gut microbiota was strongly supported through antibiotic depletion and fecal microbiota transplantation (FMT). The abolition of neuroprotection upon antibiotic treatment, and its restoration via FMT from GV-971-treated donors, underscores that an intact microbiota is essential for the drug’s action (Figure 4). Metagenomic analysis revealed that GV-971 enriched beneficial taxa within the *Duncaniella muris*, *Ligilactobacillus murinus*, *Prevotella*, *Akkermansia muciniphila*, a key producer of short-chain fatty acids that reinforce barrier function (Zhu et al., 2024; Lee et al., 2020; Peh et al., 2023). In contrast, GV-971 suppressed the expansion of *Bacillota* and *Pseudomonadota*, which can migrate to the brain via sympathetic pathways post-stroke to exacerbate infarct injury (Peh et al., 2023). While we identified these taxonomic shifts and downstream reductions in colonic cytokines, the precise molecular mediators transmitting signals from the gut to the brain warrant further characterization.

Beyond acute tissue salvage, addressing post-stroke cognitive impairment (PSCI) remains a critical unmet need (Rost et al., 2022)^.^ Our results show that GV-971 preserved neuronal density in the hippocampal CA1 and CA2 regions and significantly improved spatial learning and memory in rats (Figure 6). While Ren et al. recently elucidated a detailed molecular mechanism in a mouse MCAO/R model—linking GV-971–mediated gut microbiota remodeling to the restoration of butyrate metabolism and subsequent epigenetic regulation via HDAC3/H3K9ac/K14ac (Ren et al., 2026), our work provides critical complementary validation in a phylogenetically distinct and neuroanatomically more complex species. The use of the rat MCAO/R model, widely regarded as a gold standard in preclinical stroke research due to its higher predictive validity for human pathophysiology, confirms that the therapeutic benefits of GV-971 are not species-specific artifacts but represent a robust neuroprotective phenomenon. By characterizing both acute pathological and functional assessments at 24 h and long-term cognitive recovery after 4 weeks, our study bridges the gap between immediate post-stroke pathology and sustained functional improvement, thereby reinforcing the potential of GV-971 as a viable treatment strategy for ischemic stroke across different experimental paradigms.

Based on published literature (Mamtilahun et al., 2021), this study only used NBP as a positive control and did not explicitly compare it with GV-971, so different administration methods were used. NBP was administered intravenously to simulate the rapid systemic delivery required for acute neuroprotection in stroke patients. In contrast, GV-971 was administered orally; this choice was dictated not only by its clinical formulation but, more critically, by its mechanism of action. In addition, estradiol secreted by female rats reduced the risk of ischemic stroke; therefore, to minimize this confounding factor, female rats were not used for experimental procedures in this study and due to equipment limitations, parameters such as oxygenation and systemic physiology of postoperative rats could not be collected. Other parameters, such as the number of visits to the target hole, were not recorded. As this was an exploratory RNA-seq screening study for hypothesis generation rather than mechanistic validation, RT-qPCR or Western blotting verification was not included. Independent validation of the key molecules is warranted in future studies. This was also a limitation of our study. As a microbiota-modulating agent, GV-971 requires oral delivery to traverse the gastrointestinal tract and reshape the gut microbiota, thereby triggering a neuroprotective cascade. Furthermore, within the acute stroke setting, GV-971 serves as an oral therapeutic for long-term cognitive management.

Overall, GV-971 represents a promising multi-target strategy that overcomes key limitations of previous anti-inflammatory trials. By remodeling the gut microbiota, it simultaneously dampens central glial activation, restores BBB integrity, suppresses peripheral inflammation, and improves cognitive outcomes. These findings validate the gut–brain axis as a tractable target for stroke therapy and these preclinical findings provide preliminary experimental evidence for further evaluating the potential of GV-971 in stroke treatment. GV-971 as a strong candidate for clinical translation, is more likely to be positioned as a preventive, perioperative period protective, or post-stroke restorative intervention, particularly for preventing long-term post-stroke cognitive decline.

## Conclusion

This study demonstrates that GV-971 exerts neuroprotection in ischemic stroke possibly by remodeling gut microbiota via the gut-brain axis. It significantly reduces infarct volume, attenuates gliosis, preserves blood-brain barrier integrity, and improves cognitive function. These findings indicate that GV-971 demonstrates neuroprotective potential in experimental ischemic stroke and post-stroke cognitive impairment. Further mechanism and translational studies are needed before clinical application.

## Data Availability

The data presented in the study are deposited in the NCBI Sequence Read Archive (SRA) database under the BioProject accession number PRJNA1494104.

## Supplementary material

SUPPLEMENTARY FIGURE S1 RNA-Seq Analysis of Brain Tissue in the Injured Hemisphere. **(A)** Brain tissue RNA-Seq volcano plot. **(B)** Brain tissue RNA-Seq KEGG enrichment analysis.

SUPPLEMENTARY FIGURE S2 Fecal Metagenomic KEGG and GO Enrichment Analysis. **(A)** Fecal metagenomic KEGG KO enrichment analysis. **(B)** Fecal metagenomic KEGG Module enrichment analysis.

SUPPLEMENTARY FIGURE S3 Western blot original bands of Figures 2I–K. Supplement 3: shows the whole blot after cutting membrane at molecular weight: ZO-1(220 kDa), Occludin(65 kDa), β-actin(42 kDa).

SUPPLEMENTARY FIGURE S4 Short chain fatty acids in feces. Supplement 4: Short chain fatty acids in feces. **(A)** Acetic acid in feces. (n = 5). **(B)** Butyric acid in feces. (n = 5). **(C)** Propionic acid in feces. (n = 5). Data were presented as mean ± sem. Statistical method: one-way ANOVA. ^#^ *P* < 0.05 versus MCAO.
