# Supplementary figures and images for: Sodium oligomannate reduces cerebral infarction and improves neurological function through microbiota remodeling in MCAO/R rats
Source: Front Pharmacol. 2026 Jul 17;17:1880590. doi: 10.3389/fphar.2026.1880590 (PMC13423871; doi:10.3389/fphar.2026.1880590)

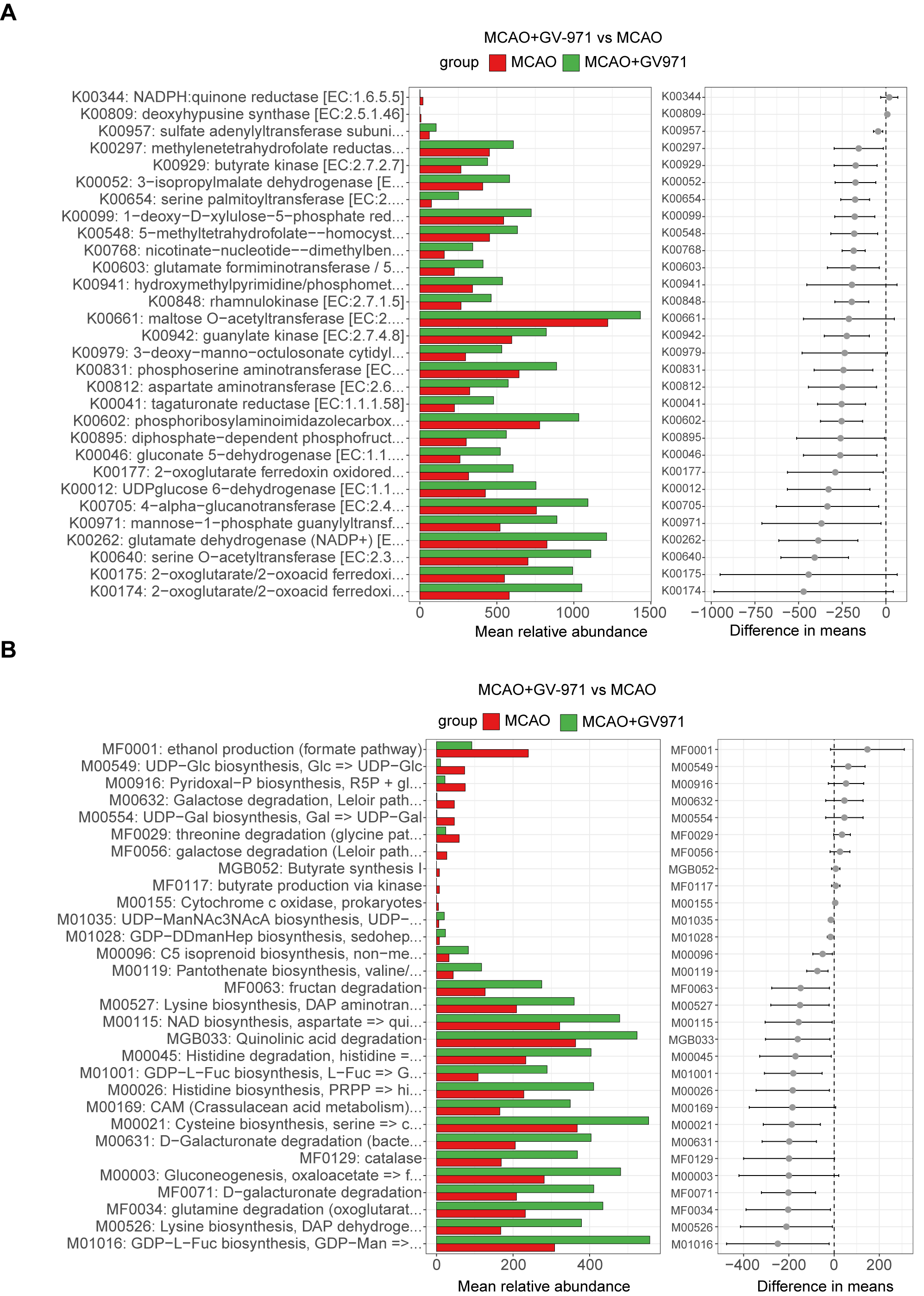

Supplement: Supplementary file 1 [file Image3.tif]

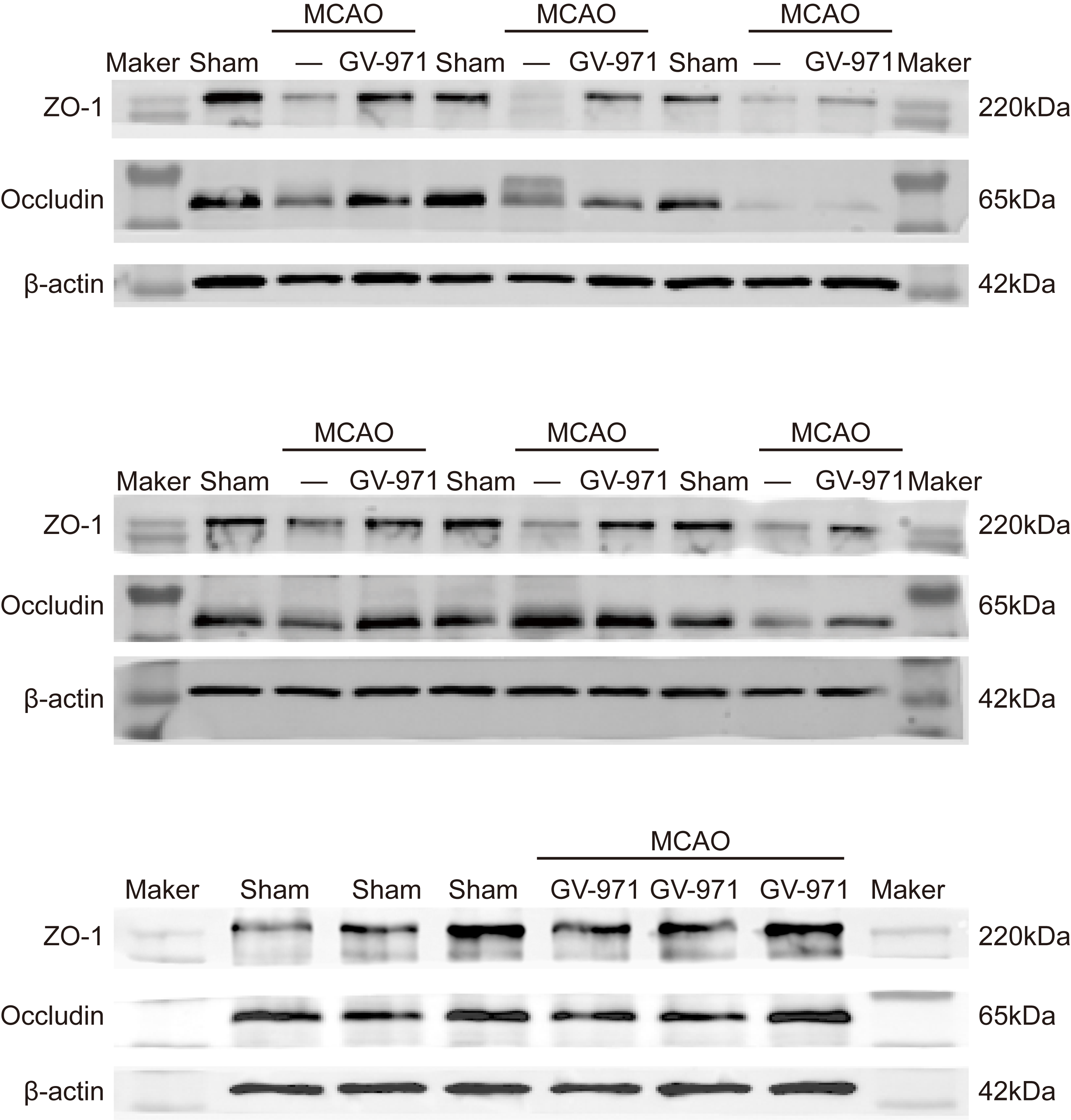

Supplement: Supplementary file 2 [file Image4.tif]

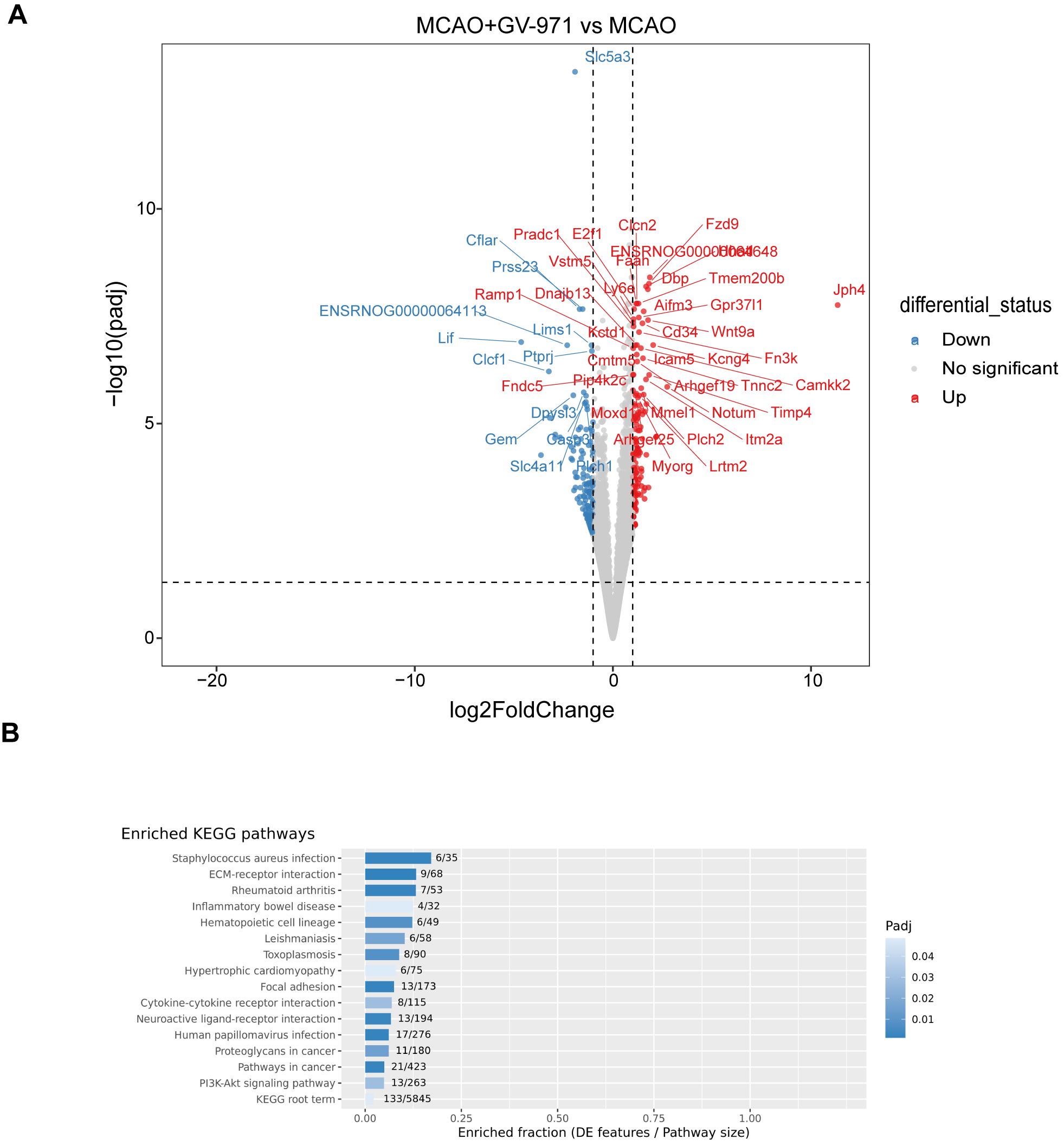

Supplement: Supplementary file 3 [file Image2.tif]

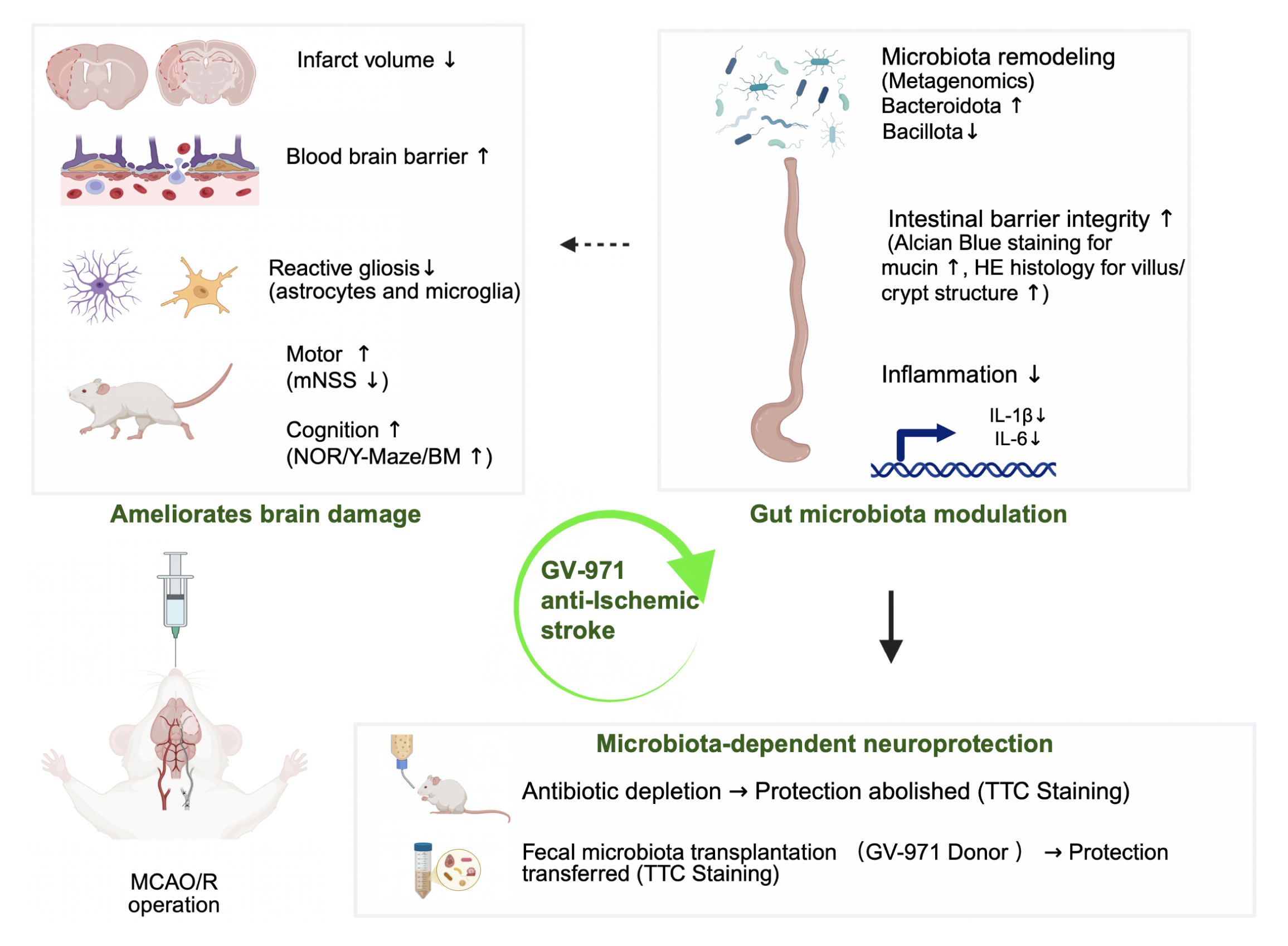

Supplement: Supplementary file 4 [file Image1.tif]

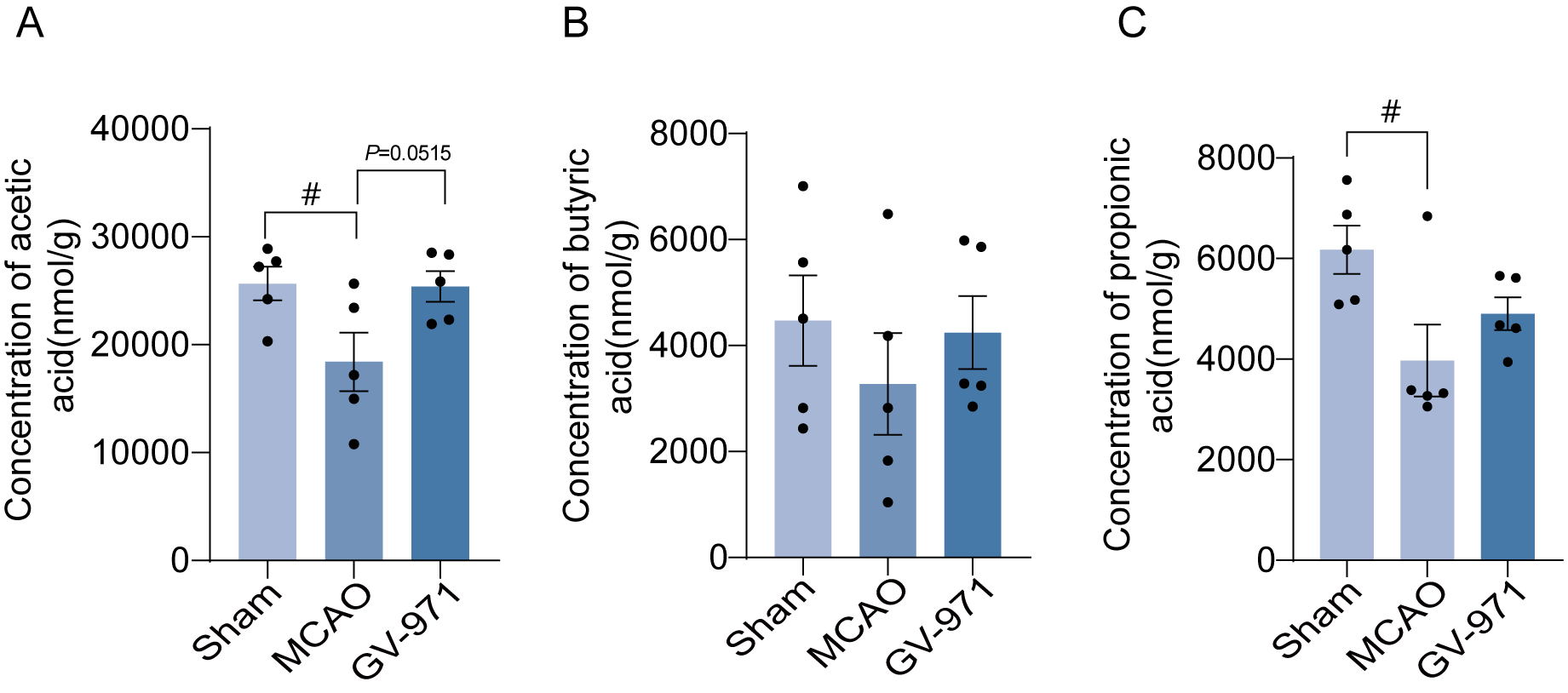

Supplement: Supplementary file 5 [file Image5.tif]
